# Sugar Coating: Utilisation of Host Serum Sialoglycoproteins by *Schistosoma mansoni* as a Potential Immune Evasion Mechanism

**DOI:** 10.3390/pathogens11040426

**Published:** 2022-03-31

**Authors:** Maude Dagenais, Jared Q. Gerlach, Timothy G. Geary, Thavy Long

**Affiliations:** 1 Institute of Parasitology, McGill University, Ste-Anne-de-Bellevue, QC H9X 3V9, Canada; timothy.g.geary@mcgill.ca (T.G.G.); thavy.long@mcgill.ca (T.L.); 2 Advanced Glycoscience Research Cluster, National University of Ireland-Galway, H91 TK33 Galway, Ireland; jared.gerlach@nuigalway.ie; 3 Microbes and Pathogen Biology, The Institute for Global Food Security, School of Biological Sciences, Queen’s University-Belfast, Belfast BT9 5DL, UK

**Keywords:** extracellular vesicles, exosomes, secretome, helminths, trematode, glycans, schistosomes, sialic acid, immune evasion, immunomodulation

## Abstract

Parasitic helminths resort to various mechanisms to evade and modulate their host’s immune response, several of which have been described for *Schistosoma mansoni*. We recently reported the presence of sialic acid residues on the surface of adult *S. mansoni* extracellular vesicles (EVs). We now report that these sialylated molecules are mammalian serum proteins. In addition, our data suggest that most sialylated EV-associated proteins do not elicit a humoral response upon injection into mice, or in sera obtained from infected animals. Sialic acids frequently terminate glycans on the surface of vertebrate cells, where they serve important functions in physiological processes such as cell adhesion and signalling. Interestingly, several pathogens have evolved ways to mimic or utilise host sialic acid beneficially by coating their own proteins, thereby facilitating cell invasion and providing protection from host immune effectors. Together, our results indicate that *S. mansoni* EVs are coated with host glycoproteins, which may contribute to immune evasion by masking antigenic sites, protecting EVs from removal from serum and aiding in cell adhesion and entry to exert their functions.

## 1. Introduction

Schistosomiasis, a chronic disease affecting ~240 million people worldwide and resulting in significant morbidity and mortality, is caused by infection with parasitic platyhelminths of the genus *Schistosoma* [1]. The infective stage is a short-lived larva (cercaria), which is released from freshwater snails. Cercariae infect human hosts by penetrating the skin and morphing into maturing larvae (schistosomula), which enter the host’s circulatory system and migrate to the hepatic portal system and mesenteries (*S. mansoni*, *S. japonicum*) or the venous plexus of the urinary bladder (*S. haematobium*). Larvae mature during the migration to eventually become adult male and female worms, which pair up and produce large numbers of eggs [2]. Eggs are destined to be released into the environment through faeces or urine, but many remain trapped in the liver and surrounding tissues, thereby causing tissue fibrosis and organ damage. A particularity of schistosomes is their ability to establish long-term chronic infections [2]. If left untreated, adult worms can survive within a mammalian host for many years [3]. Their longevity is attributable, in part, to their remarkable capacity to manipulate host immunity [4,5]. There is a growing need to better characterise and understand the specific and carefully orchestrated molecular dialogue employed by the parasites to modulate the host immune system, as it could lead to the identification of novel diagnostic and therapeutic targets. Parasite excreted/secreted products (ESPs) are widely regarded as key players in these host–parasite interactions with increasing attention being paid to vesicle-based secretion. We, and others, have previously reported the release of extracellular vesicles (EVs) in schistosomes [6,7,8,9,10,11,12,13]. The contents of EVs have been partially characterised in eggs [13], larvae [7], and adult schistosomes [6,8,14,15], showing enrichment in putative effectors such as proteins and microRNAs (miRNAs). It is only recently, however, that attention has been given to the carbohydrate structures (glycans) present on the surface of schistosome EVs [10,16]. There is increasing appreciation for the roles of glycans in EV biology, with reports suggesting the importance of carbohydrate moieties in interactions between EVs and target cells [10,17,18,19,20]. Despite these findings, limited information is available on schistosome EV glycomics and its role in infection [10,16].

Recently, we reported the presence of sialic acid residues on the surface of EVs released from adult *Schistosoma mansoni* in culture [16]. Moreover, we observed the bright labelling of sub-tegumental cells bodies by SNA-I, a plant-derived lectin that recognises glycan molecules with terminal sialic acid residues [16]. Sialic acids are a family of nine-carbon carboxylated sugars, which are common terminal monosaccharides of vertebrate glycans. They have mainly been identified in the animal kingdom, from the echinoderms onwards, with lower animals, including helminths, assumed to lack sialic acids [21,22,23]. We performed mass spectrometry analyses on sialylated EV and worm proteins to determine the origin of the sialylated molecules, and identified them as mammalian serum proteins. Given that, in vivo, worms reside in host venules and are thus exposed to blood proteins, this finding could indicate a novel immune evasion mechanism employed by the parasite. Incorporation of sialylated host glycoconjugates by *S. mansoni* is of great interest, as sialic acids play important roles in infection by, for example, aiding immune evasion and affecting target recognition and cell entry. Finally, we tested the antigenic profile of EV proteins and found that sialylated glycoproteins (sialoglycoproteins) did not elicit a humoral response in mice, supporting our hypothesis that sialic acid residues contribute to antigen masking.

## 2. Results

### 2.1. Lectin-Probed Western Blots and Mass Spectrometry Analysis of Reactive Bands

**Figure 1:**
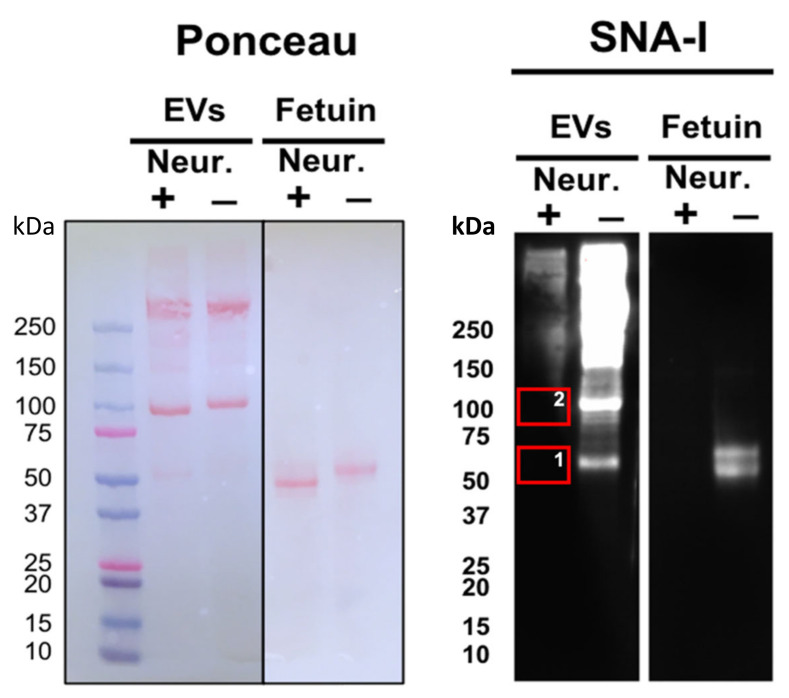
Treatment of *S. mansoni* EVs with neuraminidase (Neur) reduces SNA-I binding. EVs were incubated with a broad-spectrum neuraminidase, after which samples were resolved by electrophoresis, transferred to a PVDF membrane, and probed with SNA-I. Fetuin was used as a control sialylated glycoprotein. Ponceau-stained PVDF membrane and SNA-I-probed membrane. Two gel sections (corresponding to boxes 1 and 2) were excised and analysed by mass spectrometry.

We previously reported that several lectins exhibit strong adhesion to adult S. mansoni EVs, including the sialic-acid-binding SNA-I [16]. Treatment of EV samples with a broad-spectrum neuraminidase markedly reduced SNA-I binding compared to the strong signal observed with untreated control samples following gel electrophoresis (Figure 1), supporting our previous findings and suggesting the presence of sialylated molecules in our EV samples [16]. To determine the origin of sialic acid residues (parasite or host), we sought to identify sialylated proteins. First, we excised two of the most prominent bands (roughly 55 kDa and 100 kDa) in the neuraminidase-treated group, which strongly interacted with SNA-I in the control group (Figure 1) for analysis by mass spectrometry. We found a mixture of S. mansoni proteins and mammalian serum proteins in each of these bands, potentially suggesting a host origin of sialic acid. The mammalian serum glycoproteins included alpha-2-macroglobulin, albumin, alpha-1-antiproteinase, and Inter-alpha-trypsin inhibitor heavy chain H3 (Table 1), all of which are sialylated or have known sialylated variants [24,25,26,27,28]. Similar protein profiles were found in size-matched bands from untreated samples (data not shown), indicating that neuraminidase treatment does not affect protein content.

### 2.2. Identification of Glycosylated Proteins

To confirm that EV-associated proteins that bind SNA-I are indeed mammalian proteins, we performed a lectin pull-down assay on EV lysate. Lysed EVs were incubated with biotinylated SNA-I-coated beads, and MS spectra were searched against the Schistosoma mansoni, Mus musculus, and Bos taurus Uniprot databases. This experiment pulled down a total of 20 mammalian proteins, 15 of which were bovine serum proteins (Table 2). These include alpha-2-macroglobulin, alpha-1-antiproteinase, clusterin, hemoglobin, bovine complement proteins, and alpha-2-HS-glycoprotein (Table 2). The five other proteins identified, actin, talin, glyceraldehyde-3-phosphate dehydrogenase, vitronectin, and filamin, have all previously been identified in exosomes from various species [6,29,30,31,32].

### 2.3. Sialylated EV Proteins Do Not Elicit a Humoral Response in the Murine Host

**Figure 2:**
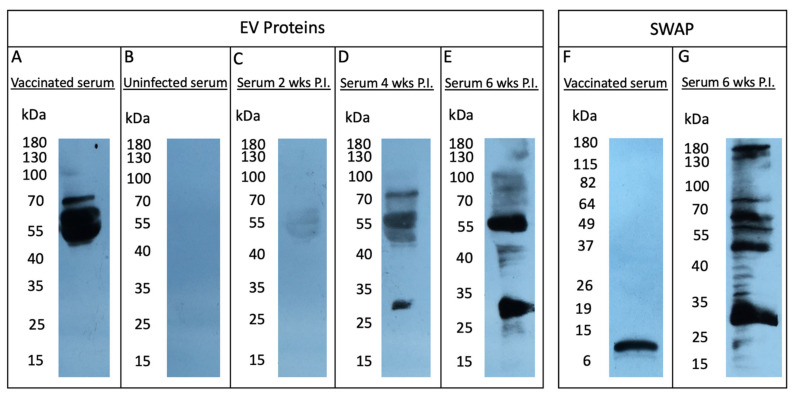
*S. mansoni* EVs and SWAP were prepared as detailed in the Materials and Methods section. The antigenicity of EV proteins was tested on EVs separated by SDS-PAGE, and Western blot (WB) analysis was performed with (**A**) serum from mice vaccinated with EVs, (**B**) serum from uninfected mice, or serum from infected mice collected at (**C**) 2 weeks, (**D**) 4 weeks, or (**E**) 6 weeks post-infection (P.I.). SWAP proteins were probed using serum from (**F**) vaccinated mice or (**G**) infected mice (6 weeks P.I.).

To investigate our hypothesis that EV-associated mammalian sialoglycoconjugates contribute to antigenic escape (e.g., antibody recognition), we assessed the antigenic profile of *S. mansoni* EV proteins by Western blot analysis (Figure 2) and compared these findings with the profile of sialylated proteins (Figure 1). We probed EV lysate with sera from mice vaccinated with *S. mansoni* EVs and detected a robust response to a cluster of three proteins with MW 50–70 kDa (Figure 2A). We next tested if *S. mansoni* infection induced similar antibody responses to EV proteins by probing EV lysate with serum from infected animals at 2, 4, and 6 weeks post-infection (Figure 2C–E). Interestingly, a different profile of EV proteins was recognised by serum from infected animals, with the most reactive proteins ranging between 30 and 55 kDa (Figure 2C–E). For comparison, Schistosome soluble worm antigenic preparation (SWAP) proteins were also probed with serum from infected mice 6 weeks post-infection, which revealed a similar band at 30 kDa, but otherwise displayed a different immunogenic profile (Figure 2G). To test the capacity of anti-EV antibodies to recognise schistosomal proteins, SWAP proteins were probed with immunised mouse serum (Figure 2F), generating one immunoreactive band at ~10 kDa and a fainter band at ~8 kDa.

Results shown in Figure 1 suggest that, with the exception of two bands at lower molecular mass (~55 and 100 kDa), the majority of sialylated EV-associated proteins migrate with molecular mass ≥ 150 kDa. These results greatly contrast with the antigenic profile observed at 2, 4, and 6 weeks post infection (Figure 2A,D,E), in which the immunoreactive proteins are generally no larger than 100 kDa. It thus appears that sialylated EV proteins induce limited or no antibody production.

## 3. Discussion

The treatment of schistosomiasis relies almost exclusively on the efficacy of a single drug, praziquantel, which is administered to tens of millions of people each year, raising concerns of drug resistance [33]. It is thus imperative to develop new treatment options for the control of schistosomiasis. An interesting avenue for the development of therapeutics is targeting the mechanisms through which parasites interact with their host. Host–parasite interactions have been shaped by thousands of years of co-evolution and are characterised by highly intricate molecular dialogues, involving the release of ESPs, such as EVs [14,34]. Helminth EVs are increasingly recognised as key mediators of host–parasite interactions, making them appealing targets for the development of new therapeutics. Thus far, helminth EV characterisation studies have mainly focused on the protein, miRNA, and lipid composition of different EV subsets, leaving the glycan content largely unexplored [6,9,14,35,36,37]. Surface carbohydrate moieties are likely required for EVs to exert their functions as they presumably are responsible for establishing contact with target cells. In fact, there is mounting evidence for a requirement of various glycan structures on both cell and EV surfaces for vesicle uptake by recipient cells [10,17,18,19,20]. We previously profiled the carbohydrate moieties coating the surface of adult *S. mansoni* EVs and reported the presence of sialic acid residues [16]. Orthologues of enzymes required for sialylation have not been found in the genomes of helminths and, for this reason, worms are generally considered unable to synthesise sialic acids [21,22]. Here, we investigated whether sialylated glycoconjugates were exogenous (host- or culture-medium-derived) or endogenous to the parasite.

Our mass spectrometry analyses revealed that the majority of EV proteins bound by the sialic-acid-specific lectin, SNA-I, were bovine serum proteins, suggesting that the sialylated components originate from the EV-depleted Fetal Bovine Serum (FBS) used for parasite culture. Previous reports of sialylation in various helminth glycoprotein preparations have systematically been attributed to contamination with host glycoconjugates [38,39,40]. However, it is important to consider that the presence of bovine sialylated serum proteins in our *S. mansoni* EV samples, despite the many washes and the density sedimentation step of our EV isolation protocol, could be relevant, given that in vivo, worms reside in host venules and are thus exposed to similar blood proteins. In addition, previous analysis of our samples revealed high abundance of *S. mansoni* proteins and no evidence of murine or bovine miRNAs [6], suggesting minimal contamination of *S. mansoni* EVs with host or bovine EVs occurring during our EV isolation protocol, if any. A possible interpretation of these results is that *S. mansoni* EVs are coated in vivo with host glycoproteins, which may serve to protect EVs from removal from serum and aid in cell adhesion and entry to exert their functions. Our findings might thus be indicative of a novel immune evasion and/or immunomodulation mechanism employed by the parasite, in which schistosomes EVs are coated with exogenous host-sialylated molecules. Our results suggest that most sialylated EV-associated bovine proteins do not elicit antibody production in mice under these conditions, which is consistent with the idea that the coating of EVs by sialoglycans could contribute to immune evasion by masking antigenic sites.

Sialic acids, predominantly terminating the ends of many vertebrate glycans, play key roles in cell communication and cell adhesion, and are important regulators of the immune system [23,41]. Sialoglycans have been shown to affect immune responses in many ways. Sialic acids are believed to serve as self-markers, aiding in the discrimination between self and non-self in order to prevent autoimmune activity [23]. In addition, sialic acids have the ability to mask recognition sites via electrostatic repulsion and/or steric hindrance [41,42]. In that context, aberrantly high expression of sialoglycans is a long-recognised tumour evasion strategy and is a common characteristic of tumour cells, protecting malignant cells from recognition and thus removal by immune cells [43,44]. It was initially suggested that the thick layer of sialoglycans on the surface of tumour cells concealed surface antigens, preventing immune recognition. However, increasing evidence suggests that sialic acids exert their immunomodulatory functions via various mechanisms, such as interactions with sialic-acid-binding immunoglobulin (Ig)-like lectins (Siglecs), which are expressed on the majority of leukocytes and are characterised by an N-terminal Ig domain that recognises sialylated glycans [45]. Siglecs also often contain at least one immunoreceptor tyrosine-based inhibitory motifs (ITIMs) at their C-terminus, which, once activated, inhibit cellular activation [46]. Hence, most sialic acid–Siglec interactions result in dampening of immune responses and inhibition of immune cells and are thus believed to contribute to the distinction between self and non-self [47]. Moreover, surface sialic acids prevent complement activation by recruiting and binding the complement control molecule factor H, obstructing C3b binding, and thus blocking activation of the alternative complement pathway [47,48]. The highly negative charge resulting from hypersialylation of cell surfaces also likely affects cellular interactions. For example, charge repulsions are believed to hinder the formation of immunological synapses between tumour cells and natural killer (NK) cells, preventing NK-cell-driven cytotoxicity [45].

Importantly, many pathogens have evolved the ability to coat themselves with sialic acid, aiding in evasion of the host immune response and the ability to interact with and invade host cells [49,50]. Examples include some viruses [51], bacteria [52], pathogenic fungi [53], and protozoa [50]. While some microorganisms, such as fungi and some bacteria, are capable of de novo synthesis of sialic acid, other bacteria and protozoa have to rely on acquisition from exogenous sources [47,54]. Pathogens which acquire sialic acids from the environment (e.g., the host) do so via a number of ways. Many pathogenic bacteria, for instance, express a sialidase, which cleaves terminal sialic acid from host sialoglycans, or a trans-sialidase, which hydrolyse sialic acids and directs their transfer onto bacterial structures [52,55]. However, there are examples of sialic-acid-utilising bacteria lacking sialidase-encoding genes [56], which are hypothesised to be reliant on sialidase activity either from the host [57,58] or other sialidase-encoding bacteria from the same niche [59].

Trypanosomes are well-known examples of trans-sialylation, whereby the parasites acquire host sialyl residues using trans-sialidase enzymes, allowing them to transfer terminal sialic acids from host glycoconjugates onto the terminal galactose residue of their own asialoglycoconjugates (non-sialylated glycoconjugates). This “theft” of sialic acid is well-documented in *Trypanosoma cruzi*, in which this trans-sialidase activity confers protection to the parasite, and aids adhesion to and invasion of host cells [50,60,61,62,63].

Our results suggest that schistosomes might employ a similar strategy for co-opting host sialic acid, differing, however, by their apparent use of sialylated host serum proteins as opposed to carbohydrate residues alone. The underlying mechanisms remain unknown and are subjects deserving of further investigation. It could prove interesting to examine whether *S. mansoni* EVs interact with Siglecs, which could contribute to their internalisation as is the case for some sialic acid-coated viruses that utilise Siglec-1 binding on the surface of macrophages to invade host cells [51,64]. It will also be valuable to investigate the effects of sialic acid removal on schistosome EV cell entry and effector functions.

This work provides the first indication of the use of sialic acid by parasitic helminths, offering critical insights into parasite biology as well as a potential new avenue for the development of therapeutics. It will be important, however, to confirm that this phenomenon is also observed in vivo as our observations are based on in vitro culture. Further experimental approaches are required to understand the mechanism of sialic acid acquisition and the roles of this coating strategy.

## 4. Materials and Methods

### 4.1. Parasites

Adult *S. mansoni* were obtained by cardiac perfusion of infected female CD1 mice 7 weeks after infection as per approval from the McGill University Animal Care Committee (Permit # 2019–8138). Parasites were cultured as previously described [6,16]. Briefly, worms were washed 3 times in sterile RPMI-1640 for 15 min and subsequently maintained in RPMI-1640 medium supplemented with 100 U penicillin, 100 mg/mL streptomycin, and 10% EV-depleted FBS (Life Technologies, Burlington, ON, Canada; Ref: 16000). EV depletion was performed in advance by ultracentrifugation of FBS under sterile conditions for 18 h at 120,000× *g* and 4 °C, followed by filtration through 0.22 μm hydrophilic PVDF Durapore membranes (EMD Millipore, Billerica, MA, USA; SVGV01015) [65].

### 4.2. Isolation of Adult Schistosoma mansoni Extracellular Vesicles

EVs were isolated as previously described [6,16]. Briefly, parasite-conditioned culture medium was collected after 48 and 72 h of culture and centrifuged at increasing speeds, 300× *g*/15 min, 700× *g*/15 min, and 3000× *g*/15 min, to remove larger debris, following which the supernatant was centrifuged at 12,000× *g* for 45 min. Supernatants were filter-sterilised using a 0.22 µm hydrophilic PVDF Durapore membrane (EMD Millipore; SVGV01015) and centrifuged at 100,000× *g* for 2 h using a Beckman-Coulter SW-48 rotor on a Beckman-Coulter ultracentrifuge. The pellet was re-suspended in sterile cell culture grade GIBCO^®^ Dulbecco’s Phosphate-Buffered Saline (DPBS; Life Technologies) and loaded onto a sucrose step gradient (25%, 30% and 35%), then centrifuged at 120,000× *g* for 18 h using an SW-48 rotor. Following centrifugation, the 30% sucrose fraction was collected and washed by diluting 4–5-fold with sterile DPBS, and EVs were re-pelleted by centrifugation in an SW-28 rotor at 100,000× *g* for 2 h. Pellets were washed by re-suspension in DPBS and centrifuged in an SW-41 rotor at 100,000× *g* for 2 h. Finally, pellets were re-suspended in 1.0 mL of DPBS, transferred to 1.5 mL Beckman-Coulter ultracentrifuge tubes and centrifuged in an Optima TL100 tabletop ultracentrifuge (Beckman-Coulter, Mississauga, ON, Canada) for 1.5 h at 100,000× *g* using a TLA-100.3 rotor. EVs were quantified based on protein concentration using a Pierce BCA Protein Assay Kit (ThermoFisher Scientific, Burlington, ON, Canada) and absorbance-measured at 562 nm using a Synergy H4 Hybrid plate reader. The pelleted material was snap-frozen and stored at −80 °C until use.

### 4.3. Neuraminidase Treatment

All neuraminidase treatments were carried out using a broad-spectrum α2–3,6,8,9 Neuraminidase A (New England Biolabs P0722, Ipswich, MA, USA). Proteins (50 μg EV samples) were incubated with 40 units (2 μL) neuraminidase overnight at 37 °C according to the manufacturer’s protocol and assayed immediately. 10 μg fetuin (New England Biolabs P0722, Ipswich, MA, USA) was used as a control sialylated glycoprotein.

### 4.4. Lectin-Probed Western Blot Assay and Extraction of Reactive Bands

Samples were mixed with Laemmli buffer (4% SDS, 20% glycerol, 10% 2-mercaptoethanol, 0.004% bromophenol blue, and 0.125 M Tris HCl) and incubated at 95 °C for 5 min. Aliquots were resolved by electrophoresis through 4–15% Mini-PROTEAN^®^ TGX™ Precast Protein Gels (Bio-Rad, Mississauga, ON, Canada) and transferred to a PVDF membrane according to standard protocols. Five μg fetuin (New England Biolabs P0722, Ipswich, MA, USA) was used as a control sialylated glycoprotein. Blots were blocked overnight at 4 °C in blocking buffer (1X Tween 20 (TBST), 0.1% Tween 20, 5% BSA) and probed with biotinylated SNA-I (1:200 in PBS; Biolynx B-1305, Brockville, ON, Canada), after which they were incubated with streptavidin–horseradish peroxidase (1:5000 in blocking buffer; abcam ab7403, Cambridge, MA, USA). Blots were developed using Pierce™ ECL Western Blotting Substrate (Thermo Fisher Scientific, San Jose, CA, USA) following the manufacturer’s protocol and imaged with a ChemiDoc MP Imaging System (Bio-Rad). For mass spectrometry analysis, freshly run gels were rinsed 3 times for 5 min with 100 mL of deionised water and stained by immersion in SimplyBlue™ SafeStain (Life Technologies, Burlington, ON, Canada) for 1 hr at room temperature with gentle shaking. The staining solution was discarded and the gel washed with 100 mL of water for 2 h. The two most prominent bands that lost reactivity with SNA-I in neuraminidase-treated samples were excised in the neuraminidase-treated group and de-stained with 30% ethanol. Bands were rinsed in ultrapure water and processed and analysed by MS/MS at the Plate-forme protéomique CHU de Quebec as described below.

### 4.5. Lectin Pulldown

Streptavidin-conjugated resin beads (Novagen 69203–3, San Diego, CA, USA) were incubated with 250 μg biotinylated lectin SNA-I for 1 h on a rotating incubator at 4 °C. Beads were washed 10X with PBS in Pierce™ Spin Columns (ThermoFisher Scientific, 69705). Lectin-beads were incubated with lysed EVs. Briefly, 155 μg EVs were lysed in PBS, 0.1% Triton X-100, diluted 10-fold in PBS and centrifuged at 3000× *g* for 3 min. The supernatant was collected, added to the SNA-I-conjugated beads and incubated overnight at 4 °C on a rotating incubator. Beads were washed 10 times with PBS followed by 5 washes with 50 mM ammonium bicarbonate (pH 7.4) and sent to the CHU de Quebec Research Center for mass spectrometry analysis.

### 4.6. Protein Digestion

Protein digestion and mass spectrometry experiments were performed by the Proteomics platform of the CHU de Quebec Research Center, Quebec, Canada. Proteins in bands extracted from gels were reduced with 10 mM DTT and alkylated with 55 mM iodoacetamide. Trypsin digestion was performed using 126 nM of modified porcine trypsin (sequencing grade, Promega, Madison, WI) at 37 °C for 18 h. Digestion products were extracted using 1% formic acid and 2% acetonitrile followed by 1% formic acid and 50% acetonitrile. The recovered extracts were pooled, vacuum-centrifuge-dried and then resuspended in 10 µL of 0.1% formic acid, and 5 µL was analysed by mass spectrometry. On beads, protein digestion was carried out using 0.1 µg modified porcine trypsin (sequencing grade, Promega, Madison, WI, USA) in 50 mM ammonium bicarbonate for 5 h at 37 °C. Digestion was stopped with 5% formic acid (FA), and peptides were eluted from the beads with 60% acetonitrile (ACN) 0.1% FA. Tryptic peptides were desalted on Stage tips (Empore C18, 3 M Company), vacuum-dried, and then resuspended in LC loading solvent (2% ACN, 0.05% trifluoroacetic acid (TFA)).

### 4.7. Mass Spectrometry

Half of the amount of each sample was analysed by nanoLC/MSMS using a Dionex UltiMate 3000 nanoRSLC chromatography system (Thermo Fisher Scientific) connected to an Orbitrap Fusion mass spectrometer (Thermo Fisher Scientific) equipped with a nanoelectrospray ion source. Peptides were trapped at 20 μL/min in loading solvent (2% ACN, 0.05% TFA) on a 5 mm × 300 μm C18 pepmap cartridge (Thermo Fisher Scientific) during 5 min. Then, the pre-column was switched online with a 50 cm × 75 µm-internal-diameter separation column (Pepmap Acclaim column, ThermoFisher), and the peptides were eluted with a linear gradient from 5–40% solvent B (A: 0.1% FA, B: 80% ACN, 0.1% FA) in 30 min, at 300 nL/min (60 min total runtime). Mass spectra were acquired using a data-dependent acquisition mode using Thermo XCalibur software version 4.1.50. Full-scan mass spectra (350 to 1800 *m*/*z*) were acquired in the orbitrap using an AGC target of 4 × 10^5^, a maximum injection time of 50 ms, and a resolution of 120 000. Internal calibration using lock mass on the *m*/*z* 445.12003 siloxane ion was used. Each MS scan was followed by acquisition of fragmentation MSMS spectra of the most intense ions for a total cycle time of 3 s (top speed mode). The selected ions were isolated using the quadrupole analyzer with 1.6 m/z windows and fragmented by Higher energy Collision-induced Dissociation (HCD) with 35% of collision energy. The resulting fragments were detected by the linear ion trap at a rapid scan rate with an AGC target of 1 × 10^4^ and a maximum injection time of 50 ms. Dynamic exclusion of previously fragmented peptides was set for a period of 30 s and a tolerance of 10 ppm.

### 4.8. Database Searching

MGF peak list files were created using Proteome Discoverer 2.3 software (Thermo). MGF files were then analysed using Mascot (Matrix Science, London, UK; version 2.5.1). Mascot was set up to search a contaminant database and Uniprot Bos taurus (37885 entries, UP000009136), Mus musculus (63738 entries, UP000000589), and Schistosoma mansoni (16227 entries, UP000008854) databases assuming the digestion enzyme trypsin. Mascot was searched with a fragment ion mass tolerance of 0.60 Da and a parent ion tolerance of 10.0 PPM. Carbamidomethyl of cysteine was specified in Mascot as a fixed modification. Deamidation of asparagine and glutamine and oxidation of methionine were specified in Mascot as variable modifications. Two missed cleavages were allowed.

### 4.9. Criteria for Protein Identification

Scaffold (version Scaffold_4.8.4, Proteome Software Inc., Portland, OR, USA) was used to validate MS/MS-based peptide and protein identifications. A false discovery rate of 1% was used for peptide and protein. Proteins that contained similar peptides and could not be differentiated based on MS/MS analysis alone were grouped to satisfy the principles of parsimony. Peptide identifications were accepted if they could be established at greater than 95.0% probability by the Scaffold Local FDR algorithm. Protein identifications were accepted if they could be established at greater than 99.0% probability and contained at least 2 identified peptides in two independent experiments.

### 4.10. Soluble Worm Antigenic Preparation

SWAP was prepared as previously described [16]. Approximately 30 freshly perfused adult worms were freeze–thawed in 150 μL lysis buffer (1X DPBS, 0.5% Triton X-100) with cOmplete™ Mini Protease Inhibitor Cocktail (Roche, Laval, QC, Canada), homogenised using a pestle, and centrifuged at 4000× *g* for 30 min at 4 °C. The supernatant was collected and used as SWAP. SWAP concentration was determined using a Pierce BCA Protein Assay Kit as above.

### 4.11. Mouse Infection and Vaccination

CD1 mice were obtained as described above. Naïve CD1 mice received an intra-peritoneal (IP) injection of 200 μL cell culture grade PBS (Thermo Fisher Scientific) with or without 15 μg EV protein and received the same immunization regimen 2 weeks later. The experimental group and control group comprised 5 mice each. In both cases, mice were euthanised, and serum collected from blood was obtained by cardiac puncture.

### 4.12. Immunogenicity Western Blot Assays

To characterise immunogenic components, purified EVs or SWAP were mixed with Laemmli buffer [66] and incubated at 95 °C for 5 min. Aliquots were resolved by electrophoresis through a 10% acrylamide SDS-PAGE gel and transferred to a nitrocellulose (or PVDF) membrane according to standard protocols. Antigenic EV proteins were detected using control uninfected mouse serum, vaccinated mouse serum (see below), or serum from infected mice obtained 2, 4, or 6 weeks post-infection. Goat anti-mouse IgG-HRP conjugate (Santa Cruz Biotechnology, Dallas, TX, USA, sc-2354, Lot L0312) was used as the secondary antibody. Blots were developed using Pierce™ ECL Western Blotting Substrate (ThermoFisher Scientific) following the manufacturer’s protocol.

## Figures and Tables

**Table 1:** The most abundant proteins detected in excised bands 1 and 2. Bands were analysed by mass spectroscopy, and MS spectra were searched against the *Schistosoma mansoni*, *Mus musculus*, and *Bos taurus* Uniprot databases. *Bt*, *Bos taurus*.

		Total Spectrum Count	
Accession Number	Identified Proteins	Band 1	Band 2
A0A140T897	Albumin [*Bt*]	17	233
P34955	Alpha-1-antiproteinase [*Bt*]	7	62
F1MM86 (+1)	Complement component C6 [*Bt*]		13
Q7SIH1	Alpha-2-macroglobulin [*Bt*]	4	8
A0A3Q1MU98 (+1)	Complement component C9 [*Bt*]	4	
A0A3Q1NJR8	Antithrombin-III [*Bt*]		7
A0A3Q1LQ21 (+1)	Inter-alpha-trypsin inhibitor heavy chain H3 [*Bt*]		6
G3X6N3	Beta-1 metal-binding globulin [*Bt*]	3	2

**Table 2:** Proteins detected in the SNA-I pull-down experiment. MS spectra were searched against the *Schistosoma mansoni*, *Mus musculus*, and *Bos taurus* Uniprot databases. *Bt*, *Bos taurus*; *Mm*, *Mus musculus*.

UniProt Accession Number	Identified Protein	Total Spectrum Count	Unique Peptides
A2MG_BOVIN	Alpha-2-macroglobulin [*Bt*]	65	38
A1AT_BOVIN	Alpha-1-antiproteinase [*Bt*]	46	18
F1MJK3_BOVIN	Uncharacterised protein LOC506828 [*Bt*]	13	10
CLUS_BOVIN	Clusterin [*Bt*]	10	9
A0A3Q1LK49_BOVIN	Inter-alpha-trypsin inhibitor heavy chain H2 [*Bt*]	10	9
HBBF_BOVIN	Hemoglobin fetal subunit beta [*Bt*]	10	8
A0A3Q1M2B2_BOVIN	Complement C3 [*Bt*]	7	6
A0A3Q1LVV7_BOVIN	Fibrinogen alpha chain [*Bt*]	6	6
FETUA_BOVIN	Alpha-2-HS-glycoprotein [*Bt*]	6	6
A0A3Q1LQ21_BOVIN	Inter-alpha-trypsin inhibitor heavy chain H3 [*Bt*]	6	6
ACTB_BOVIN	Actin, cytoplasmic 1 [*Bt*]	6	5
A0A3Q1MLQ7_BOVIN	Talin 1 [*Bt*]	6	5
A0A3Q1LKN2_BOVIN	Thyroglobulin [*Bt*]	6	4
A0A140T881_BOVIN	Apolipoprotein E [*Bt*]	3	3
A0A3Q1LGY9_BOVIN	Angiotensinogen [*Bt*]	3	3
APOA1_BOVIN	Apolipoprotein A-I [*Bt*]	3	3
A2AP_BOVIN	Alpha-2-antiplasmin [*Bt*]	3	3
G3P_BOVIN	Glyceraldehyde-3-phosphate dehydrogenase [*Bt*]	3	3
Q3ZBS7_BOVIN	Vitronectin [*Bt*]	2	2
B7FAU9	Filamin, alpha [*Mm*]	2	2

## Data Availability

All the data presented in this study are available in this manuscript.
